# Targeting microglia microtubules: cytoskeletal remodeling as a druggable hub in neuroinflammation and neurodegeneration

**DOI:** 10.3389/fnins.2026.1812417

**Published:** 2026-04-20

**Authors:** Caterina Sanchini, Maria Rosito, Francesca Bartolini, Silvia Di Angelantonio

**Affiliations:** 1 Center for Life Nano- & Neuro-Science, Istituto Italiano di Tecnologia, Rome, Italy; 2 Department of Life Sciences, Health and Health Professions, Link Campus University, Rome, Italy; 3 Department of Pathology & Cell Biology, Columbia University Irving Medical Center, New York, NY, United States; 4 Department of Physiology and Pharmacology, Sapienza University, Rome, Italy; 5 D-Tails Research srl BC, Rome, Italy

**Keywords:** cytoskeleton, microglia, microtubules, neurodegeneration, neuroinflammation

## Abstract

Microglia dynamically remodel their cytoskeleton to surveil the brain, respond to injury, and shape synaptic connectivity. While actin drives rapid process motility and phagocytic cup formation, emerging evidence indicates that microtubules are critical regulators of microglial morphology, trafficking, and inflammatory signaling. In homeostatic microglia, microtubules are nucleated at Golgi outposts, supporting ramified architectures and low inflammatory tone. Upon activation, microglia undergo a switch to a centrosome-nucleated, radial microtubule array, driven in part by cyclin-dependent kinase 1 (Cdk1) and associated with polarized cytokine release, NLRP3 inflammasome engagement, and altered phagocytic behavior. We discuss how key regulators of this transition-including Cdk1, centrosomal γ-tubulin recruitment, Golgi-derived microtubule nucleation, and the kinase MARK4 may constitute druggable nodes to tune microglial reactivity in neuro-degenerative diseases. Finally, we outline experimental priorities for translating microglial microtubules into therapeutic targets.

## 1 Introduction

**Figure 1:**
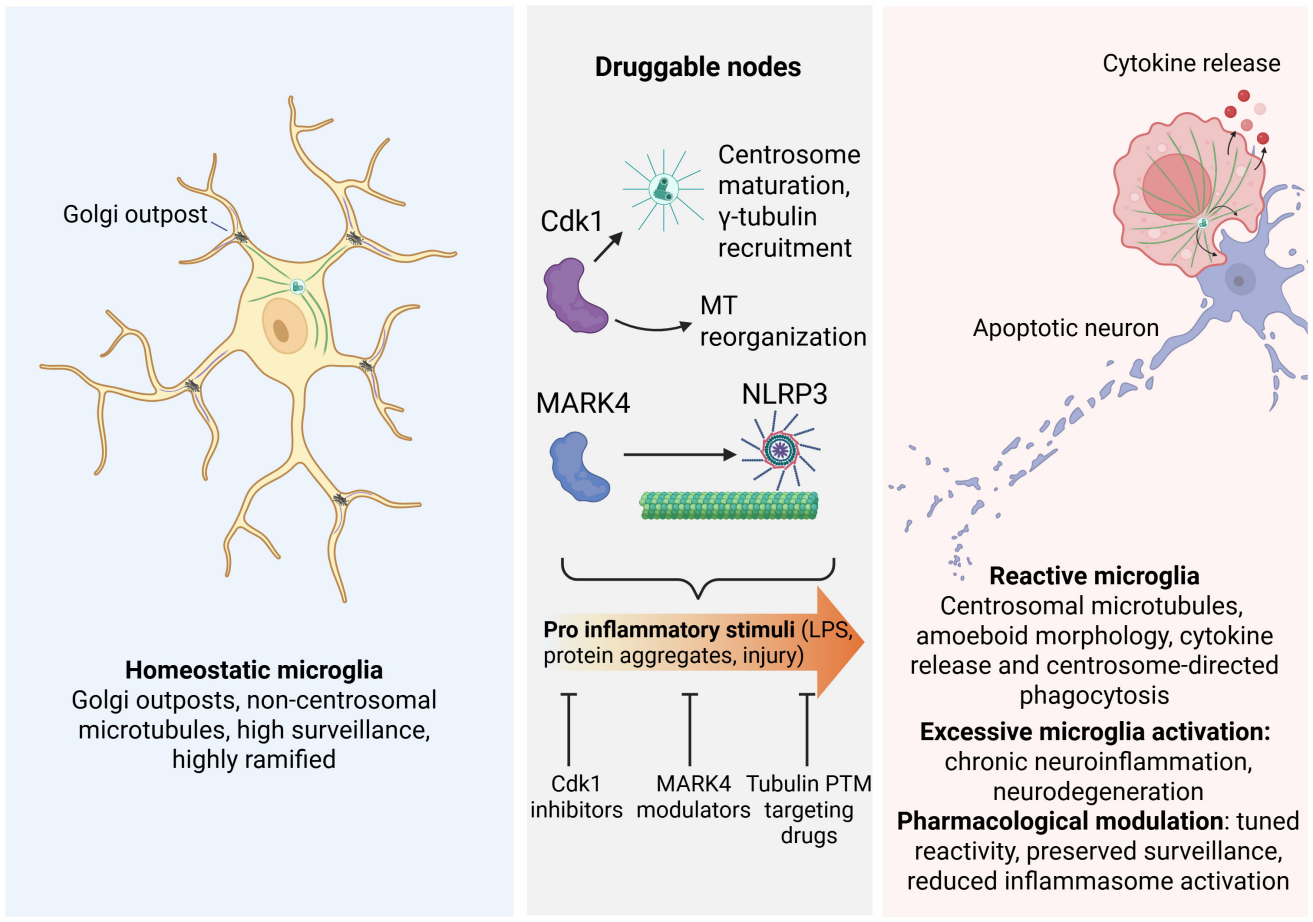
Golgi-to-centrosome microtubule remodeling in microglia and druggable regulatory nodes. Homeostatic microglia (*left*) are highly ramified and rely mainly on non-centrosomal microtubules (purple) nucleated at Golgi outposts. A minor centrosomal contribution (green) is present but does not substantially support process architecture. The central panel highlights druggable nodes: Cdk1 drives centrosome maturation, γ-tubulin recruitment, and MT reorganization, while MARK4 interacts with NLRP3 to regulate inflammasome positioning and activation. Pro-inflammatory stimuli engage these pathways and can be considered as druggable targets. Reactive microglia (*right*) adopt an amoeboid morphology with centrosomal microtubules (green), cytokine release, and centrosome-directed phagocytosis of apoptotic neurons. In this state, the centrosome (green) becomes the primary MTOC and nucleates a radial MT array, while Golgi-derived MT nucleation is reduced. (Created with BioRender.com).

Microglia are the resident immune cells of the central nervous system and continuously adapt their morphology and function to surveil the parenchyma, clear debris, and respond to injury or neurodegeneration (Colonna and Butovsky, 2017; Kettenmann et al., 2011). This plasticity relies on coordinated remodeling of the actin and microtubule (MT) cytoskeletons (Socodato and Relvas, 2024), which together control process motility, phagocytosis, and the polarized trafficking of inflammatory mediators (Davalos et al., 2005; Eyo and Wu, 2019; Ohsawa et al., 2010). While MTs and their modifications have been extensively studied in neurons and neurodegeneration (Baas et al., 2016; Janke and Magiera, 2020; Kapitein and Hoogenraad, 2015; Teoh and Bartolini, 2025), their organization and regulation in microglia have only recently begun to be elucidated (Adrian et al., 2023; Rosito et al., 2023; Socodato and Relvas, 2024) (Figure 1).

MTs are dynamic polymers of α/β-tubulin heterodimers that assemble into polarized filaments and undergo cycles of growth and shrinkage, a behavior modulated by MT-associated proteins and tubulin post-translational modifications (PTMs) (Mitchison and Kirschner, 1984; Nogales et al., 1999). These PTMs contribute to a “tubulin code” that defines long-lived, stable MT subsets and regulates organelle positioning and intracellular transport (Gadadhar et al., 2017; Janke and Bulinski, 2011; Janke and Magiera, 2020). Although this code has mainly been characterized in neurons (Moutin et al., 2021; Teoh and Bartolini, 2025), recent work demonstrates that microglia MTs also display distinct PTM patterns that correlate with their reactive state, suggesting that MT modifications encode information about microglia function (Adrian et al., 2023; Ilschner and Brandt, 1996; Rosito et al., 2023).

In homeostatic, ramified microglia, MTs form non-centrosomal arrays nucleated at Golgi outposts that support long processes and low inflammatory activity (Rosito et al., 2023). Upon stimulation, microglia undergo a striking transition to a centrosome-nucleated radial MT array, accompanied by increased MT dynamics and loss of ramified morphology (Adrian et al., 2023; Rosito et al., 2023). This switch is driven in part by cyclin-dependent kinase 1 (Cdk1) as well as associated centrosomal regulators, and has been linked to polarized cytokine release, phagocytic behavior, and inflammasome activation, positioning MT remodeling as a critical structural hallmark of microglial reactivity (Adrian et al., 2023; Li et al., 2017).

In this mini-review, we summarize current knowledge on MT organization in homeostatic and reactive microglia, highlight how the transition from Golgi-based to centrosomal MT nucleation shapes microglia function, and discuss key molecular regulators, including Cdk1, γ-tubulin recruitment, modulators of MT dynamics (such as tubulin-modifying enzymes, MT-associated proteins, and upstream kinases), and MARK4 as druggable nodes to modulate neuroinflammation in neurodegenerative disease.

## 2 Microtubule organization in surveillant microglia

### 2.1 Non-centrosomal nucleation and Golgi outposts in ramified microglia

*In vivo*, homeostatic microglia display a highly ramified morphology with thin, actin-rich filopodia and thicker processes containing both actin and MTs, which provide a stable structural backbone for continuous surveillance of the neuropil (Bernier et al., 2019; Nimmerjahn et al., 2005). Early ultrastructural studies and more recent imaging work show that MTs in ramified microglia are not organized in a classic radial array from a single centrosomal microtubule-organizing center (MTOC) but rather form parallel bundles extending through the soma and processes (Ilschner and Brandt, 1996; Rosito et al., 2023). Homeostatic microglia are characterized by a predominance of non-centrosomal MTs; a minority of centrosomal MTs may coexist, but current evidence indicates that centrosomal nucleation contributes little to process maintenance in the ramified state (Rosito et al., 2023). Upon activation, this balance is inverted, with robust γTuRC recruitment to the centrosome and assembly of a radial array that supports cytokine secretion and motility (Adrian et al., 2023; Rosito et al., 2023).

A defining feature of the homeostatic state is MT nucleation at GM130-positive Golgi outposts, which serve as extra-centrosomal MTOCs distributed along the proximal processes (Rosito et al., 2023). In other polarized cells, such as neurons, Golgi-derived MTs sustain post-Golgi trafficking, maintenance of Golgi integrity, and cell polarity, thereby directing membrane and cargo delivery toward specific cellular domains (Horton et al., 2005; Ori-McKenney et al., 2012; Valenzuela et al., 2020) to support local secretory and endolysosomal trafficking along primary and higher-order branches, stabilize branch points, and maintain the asymmetric arbor required for continuous parenchymal surveillance. In microglia, Golgi-derived MTs could provide dedicated tracks for the polarized delivery and recycling of receptors, ion channels, and signaling complexes at motile tips, as well as for the targeted transport of phagosomes and degradative organelles during low-level, homeostatic clearance. This organization is associated with mixed MT polarity and enhanced process complexity, and functionally correlates with low cytokine output, supporting the notion that Golgi-based MT nucleation acts as a structural signature of a surveillant, low-reactivity state (Bernier et al., 2019; Rosito et al., 2023). We therefore speculate that Golgi-associated MT nucleation may not only affect microglia morphology but also couple local trafficking with branch geometry, promoting the efficiency and spatial fidelity of surveillance. Because changes in MT organization are expected to precede gross morphological retraction or robust cytokine production in reactive microglia, MT-based “early cytoskeletal readouts” - including loss of Golgi-derived MTs, altered branch-associated PTM patterns, or redistribution of Golgi outposts - could serve as proximal biomarkers of microglial priming and as pharmacodynamic endpoints to monitor interventions aimed at preserving or restoring surveillant microglia.

### 2.2 Tubulin post-translational modifications and microglia surveillance

Tubulin PTMs, including acetylation and detyrosination, are widely recognized as markers of long-lived MTs that preferentially interact with specific motor proteins and cargoes, are linked to resistance to depolymerizing agents as well as establishment of cell polarity (Gadadhar et al., 2017; Janke and Magiera, 2020; Moutin et al., 2021). The tubulin PTM landscape may have several implications for microglia surveillance, reactivity and their monitoring. First, stable, PTM-rich MTs likely facilitate efficient organelle transport and positioning of endolysosomal compartments along processes, supporting continuous sampling and phagocytic capacity in the absence of full activation (Bernier et al., 2019; Nimmerjahn et al., 2005; Rosito et al., 2023). Second, the relative abundance and spatial distribution of acetylated and detyrosinated tubulin may act as quantitative biomarkers of microglial state, providing a cytoskeletal readout that complements classical molecular markers of homeostasis or reactivity (Adrian et al., 2023; Bennett et al., 2016; Butovsky et al., 2014; Rosito et al., 2023). Finally, because enzymes that write and erase these PTMs are drug-targetable, the tubulin code of microglial MTs offers an attractive avenue to preserve or restore a surveillant phenotype by modifying homeostatic MT subsets. Measurements of tubulin PTMs in activated microglia, however, show conflicting evidence. While (Adrian et al. 2023) observed an increase in acetylated tubulin after LPS treatment, (Rosito et al. 2023) reported reduced acetylated and detyrosinated tubulins, accompanied by decreased MT dynamicity, suggesting decreased MT stability. Similarly, (Ilschner and Brandt 1996) described reduced levels of stabilizing PTMs in amoeboid microglia, supporting increased MT dynamics during activation. Rather than representing true contradictions, these discrepancies likely reflect both methodological differences (fixation protocols, antibody specificity, stimulation paradigms) and biological heterogeneity. Distinct activation models (acute vs. chronic stimuli, peripheral vs. CNS-derived challenges, different inflammatory contexts) may differentially engage tubulin-modifying enzymes, producing PTM patterns that are specific to inflammatory phase or disease context. Moreover, transcriptional and functional diversification into context-dependent microglia subsets suggests that tubulin PTM signatures may vary across microglia states, an aspect that remains largely unexplored but is critical for interpreting apparently conflicting datasets. Overall, these findings underscore that MT stabilization and PTM dynamics during microglial MT remodeling require further systematic investigation.

## 3 Microtubule organization in activated microglia

### 3.1 Centrosome-nucleated radial arrays and morphological transformation

Upon inflammatory stimulation, microglia undergo a profound reorganization of their MT cytoskeleton, transitioning from non-centrosomal, Golgi-based arrays to a centrosome-nucleated radial network that parallels the shift from ramified to amoeboid morphology (Adrian et al., 2023; Gadadhar et al., 2017; Rosito et al., 2023). In LPS- and IFN-γ-treated microglia, γ-tubulin and pericentriolar material redistribute from Golgi outposts to the centrosomal region, which becomes the main MTOC and nucleates MTs radiating throughout the cytoplasm. This structural switch has functional consequences. Centrosome-anchored radial MT arrays facilitate polarized trafficking between the perinuclear region and nascent protrusions, favoring cytokine secretion and directed migration of reactive microglia (Adrian et al., 2023; Nimmerjahn et al., 2005). *In vivo*, similar centrosomal clustering of MT-nucleating components has been observed in microglia during retinal inflammation, suggesting that pericentrosomal accumulation of γ-tubulin and loss of Golgi-derived MTs represent conserved cytoskeletal hallmarks of microglial reactivity (Rosito et al., 2023). These features position centrosome-driven MT nucleation as a structural node that could be pharmacologically targeted to restrain or reprogram microglial activation without entirely abolishing their surveillance capacity. At both the Golgi and the centrosome, MT nucleation typically relies on the γ-tubulin ring complex (γTuRC), a conserved multi-subunit complex that templates α/β-tubulin assembly (Liu et al., 2020). In homeostatic microglia, MT arrays display mixed polarity (Rosito et al., 2023; Adrian et al., 2023), reminiscent of dendrites and other highly polarized cells, suggesting that local positioning and orientation of Golgi outposts and/or γTuRC-containing nucleation modules along processes may bias the direction of MT plus-end growth and thereby shape branch architecture and trafficking routes. Work in neurons shows that γTuRC and associated factors, including the augmin complex, support non-centrosomal nucleation from existing MTs and from Golgi outposts, contributing to local MT density and polarity without a single dominant MTOC (Mukherjee et al., 2024; Sánchez-Huertas et al., 2016; Zhang et al., 2024). By analogy, γTuRC-augmin modules in microglia could branch MTs from process-associated MTs or from Golgi outposts to generate dense, mixed-polarity arrays that stabilize ramified arbors and support local secretory and endolysosomal trafficking. The precise contribution of γTuRC components and augmin to MT organization in microglia is currently unknown and should be investigated, for example by targeted perturbation of these components in homeostatic versus reactive microglia.

### 3.2 Cdk1 as an upstream regulator and druggable kinase

Adrien et al. have identified cyclin-dependent kinase 1 (Cdk1) as a key upstream regulator of this activation-induced MT remodeling in microglia (Adrian et al., 2023). In primary murine microglia, LPS stimulation induces Cdk1 activation, which promotes centrosomal maturation, enhances γ-tubulin recruitment, and drives the reorganization of MTs into a radial array that supports amoeboid morphology and pro-inflammatory output. Mechanistically, Cdk1 phosphorylates and downregulates the MT-destabilizing protein Stathmin 1 while enhancing the activity of the MT associated protein Map4, a process that may shift the balance of MT dynamics toward a configuration that favors reactive behavior (Adrian et al., 2023; Fourest-Lieuvin et al., 2006).

Pharmacological inhibition of Cdk1 using the selective small-molecule inhibitor RO-3306 attenuates LPS-induced morphological transformation, preserves more ramified features, reduces centrosome-dependent MT polymerization, and diminishes cytokine release in a dose-dependent manner. Similar but less specific effects are observed with broad Cdk inhibitors such as roscovitine, supporting the notion that Cdk activity is a central driver of MT remodeling during microglial activation (Adrian et al., 2023). Although limitations in the pharmacokinetic properties of current Cdk1 inhibitors have so far hampered *in vivo* validation, these findings highlight Cdk1 as a promising druggable kinase at the interface between MT organization, microglial morphology, and inflammatory signaling, and they provide a conceptual framework for designing more selective or brain-penetrant regulators of this pathway to modulate neuroinflammation.

## 4 Microglia microtubules in phagocytosis and inflammasome signalling

### 4.1 Centrosome positioning and serial phagocytosis

Beyond their role in global morphology, MTs and the centrosome orchestrate microglial phagocytic behavior. Live imaging in zebrafish revealed that microglia extend multiple MT-based branches that simultaneously contact several apoptotic neurons, yet typically only one target is fully engulfed at a time (Möller et al., 2022). Successful efferocytosis is predicted by the targeted translocation of the centrosome into the branch that completes phagosome closure, whereas branches that fail to recruit the centrosome often abort engulfment (Möller et al., 2022; Stötzel and Kiermaier, 2022). Experimentally doubling the number of centrosomes in microglia increases the rate of engulfment and allows concurrent phagocytosis of two neurons, demonstrating that centrosome positioning is a rate-limiting factor for branch-mediated efferocytosis (Möller et al., 2022). These observations further underscore that the centrosome, acting as the principal MTOC in reactive microglia, not only shapes MT architecture but also allocates phagocytic capacity among competing branches.

MT-guided centrosome translocation in microglia thus emerges as a structural gatekeeper of serial phagocytosis, with potential implications for how microglia prioritize targets in crowded pathological environments such as neurodegenerative lesions (Möller et al., 2022; Stötzel and Kiermaier, 2022). Modulating centrosome dynamics or its coupling to MTs could therefore offer a means to tune microglia efferocytosis.

### 4.2 MT-dependent control of NLRP3 inflammasome via MARK4 and centrosomal trafficking

Activation of the NLRP3 inflammasome in innate immune cells, including microglia, depends on precise subcellular positioning and trafficking events that are tightly linked to the MT cytoskeleton (Li et al., 2017; Swanson et al., 2019). A pivotal player in this process is microtubule-affinity regulating kinase 4 (MARK4), which binds to NLRP3 and facilitates its transport along MTs toward the MTOC, enabling assembly of the characteristic inflammasome “speck” and optimal IL-1β production. Disruption of MARK4-NLRP3 interaction or MT-dependent transport impairs NLRP3 positioning at the centrosomal region and attenuates inflammasome activation *in vivo*, identifying MARK4 as a key cytoskeletal adaptor that couples MT organization to inflammatory output (Li et al., 2017).

Although most mechanistic data derive from macrophages, converging evidence indicates that similar MT-dependent trafficking underlies NLRP3 activation also in microglia, and that modulating MARK4 or MT stability can influence neuroinflammatory responses in models of CNS disease (Li et al., 2017; Zeng et al., 2025). These findings support a model in which MTs provide tracks that concentrate inflammasome components at a pericentrosomal hub, integrating signals from mitochondria and other organelles into a single activation platform. Targeting MARK4 or specific aspects of MT-based inflammasome transport may therefore represent a promising strategy to selectively dampen microglia NLRP3 activation without broadly suppressing microglia surveillance or other immune functions.

## 5 Therapeutic perspectives: pharmacological targeting of microglia microtubules in neurodegeneration

### 5.1 MT stabilization to dampen microglia reactivity

Further studies on MT cytoskeletal rearrangements in reactive microglia may help elucidate novel therapeutic strategies for neurodegenerative diseases. Studies in the Parkinson's disease model induced by 6-hydroxydopamine (6-OHDA) have shown that Epothilone B (EpoB), an antineoplastic agent that stabilizes MTs, besides exerting a neuroprotective role, inhibited overall microglia reactivity. Indeed, EpoB restored the levels of BV2 microglia acetylated tubulin decreased by 6-OHDA, thereby inhibiting polarization toward a pro-inflammatory phenotype and enhancing the morphological transition from an ameboid to a ramified phenotype (Yu et al., 2018). However, given the discrepancies highlighted above regarding MT stability across different microglia reactive states, and the use of microglia cell line (BV2), these results require further investigation.

MT dysfunction in microglia may also contribute to treatment-related neurological complications. Chemotherapy-induced peripheral neuropathy represents a common and debilitating side effect of several anticancer therapies, many of which target MT dynamics. For instance, paclitaxel-induced peripheral neuropathy has been shown to rely on the alteration of MT dynamics in peripheral neurons (Pero et al., 2023; Shin et al., 2021). However, this condition may also arise from the adverse drug's effects on spine-resident microglia subtypes involved in central and peripheral pain processing, including a population of spinal CD11c+ microglia involved in the remission and recurrence of neuropathic pain (Batti et al., 2016; Fan et al., 2025; Kohno et al., 2022; Zhang et al., 2025). Therefore, clarifying the contribution of microglia MT dysfunction to chemotherapy-induced peripheral neuropathy will be essential for improving therapeutic strategies.

### 5.2 Achieving microglia selectivity: off-target considerations and targeting strategies

The therapeutic promise of targeting microglia MT regulators is tempered by concerns about systemic toxicity and off-target effects, given the broad expression and pleiotropic roles of kinases such as Cdk1 and MARK4, and the widespread distribution of tubulin-modifying enzymes.

Cdk1 is a central cell-cycle kinase expressed in virtually all proliferating cells, raising legitimate concerns about effects on rapidly dividing tissues and potential neurotoxicity. However, several observations suggest that carefully titrated or context-restricted Cdk1 inhibition may be compatible with neuroprotection. Conditional Cdk1 deletion or pharmacological blockade can protect neurons in ischemic models without overtly compromising basal neuronal viability, and Cdk1 upregulation in microglia appears restricted to early proliferative phases following injury (Adrian et al., 2023). Strategies such as local CNS delivery (intrathecal, intraventricular, or convection-enhanced delivery), reactive microglia-specific promoters or AAV serotypes, or transient inhibition limited to windows of pathological proliferation may mitigate systemic exposure while exploiting the Cdk1 dependence of reactive microglia.

Tubulin PTM enzymes (acetyltransferases, deacetylases, tubulin tyrosine ligase, carboxypeptidases) are expressed across multiple tissues and regulate cytoskeletal functions in neurons, glia, and peripheral cells. Nevertheless, disease-specific tubulin PTM imbalances may be more pronounced in microglia and neurons during neuroinflammation, providing therapeutic efficacy. Tubulin PTM-selective inhibitors (e.g., HDAC6-selective inhibitors) or microglia specific AAV-driven modulators of enzyme expression that only partially normalize, rather than abolish, enzyme activity may allow fine-tuning of MT function in microglia with reduced systemic risk.

MARK4 interacts physically with NLRP3 and controls its positioning at the MTOC; genetic ablation of MARK4 selectively reduces NLRP3-dependent inflammasome activation without broadly suppressing other inflammasome pathways (Li et al., 2017). Nonetheless, MARK4 participates in other MT-associated processes, so achieving microglia-specific modulation will likely require either microglia-targeted delivery systems or allosteric modulators that preferentially interfere with the MARK4-NLRP3 interface rather than global kinase activity.

Taken together, careful dose-response studies, brain-penetrant microglia-selective formulations, and strategies that target transient or local signaling events, rather than permanent inhibition, will be essential to translate these targets into safe and effective therapies.

### 5.3 Microglia MT-based biomarkers and selective targeting strategies

The MT cytoskeleton of microglia offers several candidate biomarkers that capture early and reversible shifts in activation state. The concept of reversibility is particularly attractive from a therapeutic standpoint: if the transition from Golgi-based to centrosome-nucleated MT arrays can be pharmacologically reversed or prevented, microglia may be guided back toward a surveillant, homeostatic phenotype while preserving essential immune functions. In principle, interventions that restore Golgi-associated, PTM-rich MT subsets and dampen centrosomal nucleation could re-establish ramified morphologies, redistribute organelles and secretory pathways away from pericentrosomal hubs, and reduce inflammasome engagement without inducing overt immunosuppression. Quantitative parameters such as the ratio of Golgi-based to centrosomal MT nucleation, the abundance and spatial distribution of tubulin PTMs, and the degree of radial vs. parallel MT organization could be integrated with morphological, transcriptional, and spatial proteomic profiling to define cytoskeletal signatures of distinct microglia states and to monitor therapeutic restoration of homeostatic MT architecture across disease stages and treatment courses. Longitudinal *in vivo* imaging approaches, including intravital two-photon microscopy and advanced tissue-clearing combined with light-sheet microscopy, will be particularly powerful to validate these MT-based biomarkers as dynamic reporters of microglia state transitions and treatment responses in intact circuits. These metrics may prove particularly valuable for detecting subtle deviations from homeostasis in early neurodegenerative stages or in response to therapeutic interventions.

From a drug development perspective, pathways that specifically control microglia MT remodeling - such as Cdk1-dependent centrosomal maturation or MARK4-mediated NLRP3 transport - represent more selective targets than classical, non-discriminatory MT poisons (Adrian et al., 2023; Gadadhar et al., 2017; Li et al., 2017). Combining these molecular interventions with strategies to preserve Golgi-derived, PTM-rich MT subsets could allow fine-tuned modulation of microglia reactivity while maintaining essential surveillance and housekeeping functions. As tools for *in vivo* imaging and single-cell cytoskeletal profiling continue to advance, microglia MT-based biomarkers and druggable regulators of MT organization are poised to become central elements in the design of next-generation therapies targeting neuroinflammation in neurodegeneration.

## 6 Microglia microtubules: discussion and future directions

Microglia exhibit remarkable heterogeneity in morphology, transcriptional states, and functions, which cannot be adequately captured by simple “resting” vs. “activated” or “M1/M2” dichotomies. This complexity is reflected in their MT cytoskeleton, where homeostatic, surveillant phenotypes, disease-associated microglia, and intermediate states likely correspond to distinct but partially overlapping MT organizations, rather than to a binary switch. The transition from Golgi-based, PTM-rich MT arrays to centrosome-nucleated radial networks therefore represents one major axis of reorganization within a broader continuum of structural and functional microglia states (Adrian et al., 2023; Baxter et al., 2021; Rosito et al., 2023).

A first challenge is experimental: generating conditions that faithfully recapitulate the spectrum of MT phenotypes observed *in vivo*. Primary microglia rapidly lose homeostatic gene expression and adopt amoeboid morphologies when cultured in serum or in the absence of appropriate trophic cues, complicating the interpretation of MT changes as true activation events. The use of CSF-1 and astrocyte-conditioned media, or defined cocktails of astrocyte-derived factors, has proven essential to preserve ramified morphologies, microglia homeostatic signatures, and low basal cytokine release *in vitro*, thereby providing a more reliable baseline for studying MT remodeling upon stimulation. Human iPSC-derived microglia similarly require co-culture with neurons and astrocytes, or transplantation into brain tissue, to acquire ramified morphologies and transcriptional profiles resembling *in vivo* microglia, indicating that MT organization is tightly coupled to environmental context. Future work will benefit from systematically integrating *in vitro* and *ex vivo* MT readouts (Sanchini et al., 2023) into these improved culture systems and organoid-based models (Abud et al., 2017; Baxter et al., 2021; Bohlen et al., 2017; D'Antoni et al., 2023; Haenseler et al., 2017; Vahsen et al., 2022).

A second challenge concerns the apparent discrepancies between studies on MT stability in reactive microglia. Early work reported reduced MT stability in amoeboid microglia (Ilschner and Brandt, 1996), whereas recent studies have described both loss of stability-associated tubulin PTMs and increased MT dynamics in LPS-reactive cells (Rosito et al., 2023), as well as conditions in which MT arrays in reactive microglia appear relatively more stable than those observed in homestatic microglia (Adrian et al., 2023). Rather than representing true contradictions, these differences likely reflect the diversity of activation paradigms (e.g., LPS versus cytokines versus disease-associated ligands), timing of analysis, culture conditions, and quantitative metrics used to define “stability” vs. “dynamics.” Standardizing key parameters - such as stimulation duration and concentration, serum exposure, oxygen levels, and the combination of markers used to assess both MT organization and microglia state - will be crucial to disentangle context-dependent effects from shared core mechanisms of MT remodeling.

Methodologically, the study of microglia MTs has so far relied heavily on two-dimensional cultures and classical immunostaining. Recent advances in live-cell imaging, super-resolution microscopy, and tissue-clearing preparations open the possibility of visualizing MT architecture and centrosome dynamics in three dimensions within intact brain tissue and organoids. Combining these approaches with automated morphometric analyses and spatial transcriptomics and proteomics could enable the definition of MT-based “structural fingerprints” of microglia states, integrated with gene expression and functional readouts such as phagocytic capacity or cytokine profiles (Bobotis et al., 2024; Paolicelli et al., 2022). The development of genetically encoded MT reporters and plus-tip markers tailored for microglia in animal models will further facilitate longitudinal tracking of MT dynamics during disease progression and therapeutic interventions.

From a translational perspective, MT-targeting strategies face a double constraint. On one hand, classical MT drugs such as taxanes affect all proliferating cells and carry substantial toxicity, limiting their chronic use in neurodegenerative conditions; on the other hand, microglia share many MT regulators with neurons and other glia, raising concerns about specificity. The identification of microglia-biased nodes, such as Cdk1-dependent centrosomal maturation, or MARK4-mediated NLRP3 positioning, provides a more selective entry point to modulate MT organization and inflammatory output without globally disrupting MT function throughout the CNS (Figure 1). Nevertheless, the pleiotropic roles of kinases like Cdk1 and MARK4, and their involvement in cell cycle control and other cellular processes, underscore the need for careful dose-response studies, brain-penetrant but microglia-sparing formulations, and strategies that target transient or local signaling events rather than permanent inhibition (Adrian et al., 2023; Li et al., 2017; Rosito et al., 2023; Yang et al., 2020).

An important therapeutic implication is the potential reversibility of microglia MT remodeling. If the Golgi-to-centrosome shift represents a dynamic, stimulus-driven reorganization rather than a terminal differentiation state, interventions that restore Golgi-derived, PTM-rich MT arrays could revert activated microglia to a more surveillant phenotype, reducing inflammatory output while maintaining essential housekeeping and synaptic surveillance functions. Proof-of-concept studies with MT-stabilizing agents such as epothilone B suggest that microglia phenotype can indeed be pharmacologically modulated (Yu et al., 2018), and longitudinal imaging studies will be critical to determine the extent and kinetics of MT cytoskeletal reversibility *in vivo*.

Finally, the emerging concept of microglia MT remodeling as a “structural switch” that integrates morphology, phagocytosis, and inflammasome signaling has important implications for neurodegenerative disease. In Alzheimer's disease and other proteinopathies, where chronic microglia activation and inflammasome engagement contribute to synaptic loss and neurodegeneration, targeting MT-dependent pathways such as the Golgi-to-centrosome shift or MARK4-driven NLRP3 transport could offer a way to re-establish a more homeostatic, surveillance-oriented microglia state while preserving essential immune defenses. As tools for monitoring microglia MT organization *in vivo* improve, integrating MT-based biomarkers with functional and molecular readouts will be key to stratifying patients, tracking therapeutic responses, and ultimately validating microglia MTs as druggable hubs in neuroinflammation and neurodegeneration (Adrian et al., 2023; Li et al., 2017; Liu et al., 2019; Yu et al., 2018).

## 7 Conclusions

Microglia microtubules are emerging as central integrators of morphology, trafficking, and inflammatory signaling, placing the MT cytoskeleton at the core of how microglia sense and respond to their environment. The transition from Golgi outpost-based, PTM-rich MT arrays in homeostatic microglia to centrosome-nucleated radial networks in reactive states represents a structural switch that coordinates branching complexity, phagocytic behavior, and polarized cytokine release (Adrian et al., 2023; Rosito et al., 2023).

Recent studies have identified key molecular regulators of this switch, including Cdk1-dependent centrosomal maturation and MARK4-mediated NLRP3 positioning, which link MT remodeling to effector outputs such as cytokine secretion and inflammasome activation. These pathways, together with enzymes that write and erase tubulin PTMs, delineate a set of druggable nodes that could be targeted to tune microglia reactivity while sparing essential surveillance functions. At the same time, the ability of MT-stabilizing compounds such as epothilone B to modulate microglia phenotype and support neuronal survival in models of Parkinsonism provides a proof of principle that microglia MTs are actionable *in vivo* (Adrian et al., 2023; Andreu-Carbó et al., 2024; Li et al., 2017; Liu et al., 2019; Moutin et al., 2021; Rosito et al., 2023; Yu et al., 2018).

Going forward, integrating MT-based structural biomarkers - such as the balance between Golgi- and centrosome-derived MT nucleation, PTM patterns, and MT array geometry-with transcriptional and functional readouts will be crucial to capture the diversity of microglia states across brain regions and disease stages. Refining experimental systems, from optimized primary cultures to human iPSC-derived microglia in complex co-cultures and organoids, will be equally important to faithfully model microglia MT organization and its perturbation by candidate drugs. Together, these efforts will help translate the concept of microglia MT remodeling from a mechanistic insight into a framework for developing and monitoring therapies that target neuroinflammation in neurodegenerative diseases (Adrian et al., 2023; Bobotis et al., 2024; Haenseler et al., 2017; Liu et al., 2019; Rosito et al., 2023; Tewari et al., 2021; Vahsen et al., 2022).
